# Young cancer survivors have lower bone mineral density compared with healthy controls: a nationwide population-based study in Korea

**DOI:** 10.1038/s41598-020-57503-y

**Published:** 2020-01-20

**Authors:** Hyoeun Kim, Sunmi Yoo, Seung Guk Park

**Affiliations:** Department of Family Medicine, Inje University Haeundae Paik Hospital, Busan, (48108) Republic of Korea

**Keywords:** Osteoporosis, Risk factors

## Abstract

Direct effects of cancer cells and various cancer treatments can cause bone loss in cancer survivors. The aim of this study was to assess the risk of bone loss in Korean cancer survivors, and the relationship between body composition and bone mineral density (BMD). We hypothesized that cancer survivors would have lower BMD than healthy people, and increased muscle mass has a protective effect on BMD. We measured BMD and body composition in 259 cancer survivors (99 men and 160 women). Subjects were selected from the Korean National Health and Nutrition Survey conducted from 2008 to 2011. Body composition and BMD were measured by dual-energy X-ray absorptiometry. We examined the linear trend of lumbar BMD according to tertiles of lean mass (LM) and fat mass (FM) by linear regression, adjusting for age, alcohol consumption, smoking, exercise, 25-hydroxyvitamin D, height, protein intake, and menopausal status. Cancer survivors under 50 years of age had lower lumbar BMD compared with healthy controls (0.93 ± 0.04 g/cm^2^ vs. 1.02 ± 0.01 g/cm^2^, *p* = 0.032 in males; 0.95 ± 0.02 g/cm^2^ vs. 0.98 ± 0.01 g/cm^2^, *p* = 0.015 in females). Lumbar BMD significantly increased from the lowest to highest tertiles of LM in male (p for trend < 0.001) and marginally significantly increased in female survivors (p for trend = 0.060). In this study of Korean cancer survivors, young survivors were at higher risk of having low lumbar BMD. Higher LM had beneficial effects on BMD in cancer survivors. To prevent osteoporosis and fractures, efforts to increase lean body mass, including bone, are needed for young cancer survivors.

## Introduction

Cancer negatively affects bone health by various mechanisms. Cancer cells can directly affect the number and size of bone cells^1,2^. Cancer cells directly damage micro-arterioles and bone through inflammatory reactions^3^. Therapeutic modalities for cancer treatment, including administration of anticancer drugs, corticosteroids, aromatase inhibitors in breast cancer, and androgen deprivation therapy in prostate cancer indirectly affect bone metabolism, decrease estrogen production, and induce ovarian failure^1,3–5^. Although cancer occurs more frequently in the elderly population, patients who have experienced cancer at a young age have survived for a long time due to early diagnosis and successful treatment, and cancer survivors have increased in all ages^1,2^. Older cancer survivors experience health problems associated with cancer in addition to physiological changes from aging. Osteoporosis and fractures are examples of those health problems. With aging, bone density and muscle mass decrease, and fat mass (FM) increases. Increased mechanical loading of the musculoskeletal system such as resistance training leads to an increase in muscle size and bone density^4,6^.

Previous studies on the association between body composition and bone density in patients with cancer have focused primarily on women with breast cancer or on childhood cancer survivors^2,7,8^. Bone density change in male or Asian cancer survivors has not been a major concern. Furthermore, few studies have examined the relationship between body composition and bone density in cancer survivors, compared with the healthy population, considering age-related changes in lean mass (LM) and FM.

Therefore, our aim was to assess the increased risk of bone loss in Korean cancer survivors compared with the healthy general population, and also to evaluate the relationship between body composition and bone mineral density (BMD) in cancer survivors. In addition, we investigated the complex association between body composition and BMD in both male and female cancer survivors compared with age- and sex-matched healthy individuals who had never been diagnosed with any cancer because age and sex are major risk factors associated with bone loss.

## Methods

### Subjects

The subjects of this study were Korean individuals aged more than 19 years based on the Korea National Health and Nutrition Examination Survey (KNHANES) conducted from 2008 to 2011. The KNHANES provides representative and reliable statistics on the health and nutritional status of the Korean population through nationwide surveys. The subjects of KNHANES were selected using the multistage stratified cluster sampling method. The KNHANES was conducted by the Korea Centers for Disease Control and Prevention. The original data of the KNHANES are public information that can be downloaded from the KNHANES homepage (http://knhanes.cdc.go.kr/) after approval.

The KNHANES conducted from 2008 to 2011 provided data on BMD screening. Among 28,377 individuals who participated in the KNHANES, 802 participants reported that “I have been diagnosed with cancer from my cancer doctor”. The types of cancer were classified as stomach, liver, colorectal, lung, and thyroid cancer in both sexes, as breast and cervical cancer in women, and as prostate cancer in men. Cancer sites in anatomical areas other than those mentioned above had been classified as other sites. In the case of a benign tumor, skin cancer, carcinoma *in situ*, or cancers of unknown origin were excluded from the cancer survivor category. Participants who had chronic diseases (n = 431; diabetes mellitus, hypertension, asthma, chronic obstructive pulmonary disease, angina or myocardial infarction, stroke, or arthritis) or did not undergo dual-energy X-ray absorptiometry (DXA) (n = 233) were excluded from the study. A total of 259 individuals (99 men and 160 women) were included in the cancer survivor group. Age and sex were important covariates that affect cancer incidence, bone density, and body composition, respectively. Therefore, healthy controls were selected from the population without cancer (n = 27,575), excluding 15,400 individuals who had chronic diseases (n = 8,783) or who did not undergo DXA (n = 9,349). Then, we conducted case-control matching by randomly assigning cancer survivors and healthy controls with case-control ratios of 1:5 by age and sex. Finally, 1,295 participants (495 men and 800 women) were included in the healthy control group.

### Data collection

The survey participants visited the mobile examination center and underwent computer-assisted personal interviews regarding the health questionnaire areas, such as education, economic activities, morbidity, and medical use. A computer-assisted self-interview was conducted to assess health behaviors, such as smoking and drinking habits and mental health. The examinee underwent a health examination including physical examination (body weight, height), blood pressure measurement, blood test (serum alkaline phosphatase, 25-hydroxyvitamin D3), and BMD test at the examination center. Highly trained medical staff performed the medical interview and health examination. The staff members completed an intensive training course before the survey.

Rural residents were defined as those who lived in areas of the country other than Seoul, Gyeonggi, and six metropolitan cities. Education level was classified into less than 9 years of schooling, including compulsory primary and middle school education, and higher education. The income level was divided into quartiles based on individual income, and those in the lowest quartile income were classified as low income groups. The definition used for heavy drinking was similar to that in the KNHANES. Intake of seven or more standard drinks (men) or four or more drinks (women) for two times or more in a month was classified as heavy drinking. Sufficient physical activity was defined as walking for more than 30 minutes at least 5 times a week, performing moderate intensity activity for more than 30 min at least 5 times a week, or performing high-intensity activity for more than 20 min at least 3 times a week. We defined lack of physical activity as not meeting the recommended goal of sufficient physical activity. Current smokers were defined as those who have smoked 100 or more cigarettes in their lifetime and who currently smoke. Family history of fracture due to minor trauma or osteoporosis was evaluated. Menopausal status was determined by self-report in women.

Body weight was measured using a weighing scale (Giant 150 N; Hana Co. Ltd., Seoul, Korea) with light clothing and no shoes. Weight was measured to the nearest 0.1 kg. Height was measured using a stadiometer (Holtain Ltd, Crymych, UK) in a standing straight position with no shoes. Height was accurately recorded to the nearest 0.1 cm. Body mass index (BMI) was expressed in weight/height^2^ (kg/m^2^). BMD was measured by DXA (Discovery QDR4500W, Hologic Inc., Bedford, Massachusetts, USA) at the lumbar spine (lumbar spine 1–4) and femur (total femur, trochanter, inter-trochanter, femoral neck, Ward's triangle). The participant was laid straight on his or her back on the center of the scan table and a lumbar positioner was placed under the thigh to straighten the spine. In the measurement, 30 randomly selected samples were measured twice. The acceptable range was below 1.9% in the lumbar spine and 2.5% in the femoral neck based on the International Society for Clinical Densitometry (ISCD) recommendations. LM (g) and FM (g) were also measured using DXA. The LM used in this study included bone mass.

The nutrition survey was conducted through a face-to-face interview in the interviewee's home. Dietary intake, including daily total energy (kcal), protein (g), and calcium (mg), was estimated using 24-h recall and a food frequency questionnaire by a well-trained nutritionist. Venous blood samples were collected after overnight fasting. Blood samples were immediately stored at the appropriate temperature and transported to the Central Testing Institute in Seoul, Korea, and analyzed within 24 h. The serum 25-hydroxyvitamin D3 (25(OH)D) levels (ng/mL) were measured by radioimmunoassay (1470 Wizard gamma counter; PerkinElmer, Finland). Serum alkaline phosphatase (ALP, IU/L) level was measured using a Hitachi automatic analyzer 7600 (Hitachi, Japan).

### Statistical analysis

We compared baseline characteristics of cancer survivors and sex- and age-matched healthy controls. The chi-square test was used for categorical variables and the t-test was used for continuous variables. Data were expressed as the estimated percentage or estimated mean ± standard error. A *p*-value < 0.05 was considered statistically significant. Bone health characteristics of the participants were stratified by age, sex, and menopausal status. Significant age-related differences were observed between subgroups, and the results were compared after adjustment for age. All analyses were separately conducted for each group aged more than 50 years or less than 50 years^9,10^. Aging is a clinical risk factor for osteoporosis in men and BMD testing is recommended for men older than 50 years who had additional risk factors for fracture^9^.

We tested for a linear trend of lumbar BMD according to tertiles of LM (kg) and FM (kg) by linear regression. Subjects were divided into three groups equally distributed by 33.3% of individuals based on the tertiles of LM (kg) and FM (kg). Cut point values of tertiles of LM and FM in each group are presented. We measured linear trend by assigning LM and FM categories into a regression model as a continuous term. The potential confounders were considered including heavy drinking, current smoking, exercise, 25(OH)D level (ng/mL), height (m), daily protein intake (g/d)^11^, age (<50 years or ≥50 years)^12^, and menopausal status (premenopausal or postmenopausal, in women). Statistical analysis was performed using SPSS (version 25 for Windows, SPSS, Inc., Chicago, IL, USA). This study does not contain any identifiable personal information.

### Ethics approval and consent to participate

This study design was approved by the appropriate ethics review boards and conforms to the provisions of the Declaration of Helsinki in 1995 (as revised in Edinburgh 2000). All participants voluntarily participated in the study and provided informed consent. The Korean National Health and Nutrition Survey (KNHANES) has been approved by the institutional review board of the Korea Centers for Disease Control and Prevention (2008-04EXP-01-C, 2009-01CON-03-2C, 2010-02CON-21-C, 2011-02CON-06-C).

## Results

Characteristics of cancer survivors and sex- and age-matched healthy controls are shown in Table 1. Among the 259 cancer survivors, the mean times between a cancer diagnosis and the survey were 6.45 years (standard error = 0.66) and 6.24 years (standard error = 0.44) in men and women, respectively. The most frequently reported cancer sites in men were the stomach (n = 35, 31.1%), colorectum (n = 13, 13.8%), liver (n = 10, 13.0%), thyroid gland (n = 7, 7.9%), lung (n = 5, 5.0%), prostate (n = 4, 5.4%), and other sites (n = 25, 23.9%). The most common sites in women were the breast (n = 38, 23.2%), cervix (n = 35, 21.9%), thyroid gland (n = 34, 21.9%), stomach (n = 18, 13.4%), colorectum (n = 11, 5.5%), lung (n = 4, 1.7%), and other sites (n = 20, 12.4%). Prevalence of current smoking and heavy alcohol consumption were higher in healthy controls than in cancer survivors, while there were no significant differences in physical inactivity between the two groups. Serum ALP level was higher in cancer survivors than in healthy controls in both men and women. There were more postmenopausal women in the cancer survivor group. The mean age, education, income status, family history of fracture, BMI, serum 25(OH)D level, and daily intake of calories, protein, and calcium did not show a significant difference between the two groups (Table 1).

**Table 1:** Clinical characteristics of cancer survivors and sex- and age- matched healthy controls.

	Male			Female		
	Cancer survivors (n = 99)	Healthy controls (n = 495)	p*-*value	Cancer survivors (n = 160)	Healthy controls (n = 800)	p-value
Age (years)	58.13 ± 1.89	57.11 ± 0.96	0.621	49.05 ± 0.96	49.03 ± 0.42	0.989
Rural residence (%)	31.6	33.9	0.704	29.3	26.4	0.466
Duration of education ≤9 years (%)	42.1	46.7	0.486	36.1	37.2	0.812
Low income (%)	26.5	23.7	0.640	24.9	21.5	0.420
Heavy drinking (%)	33.2	47.2	0.039	8.9	17.4	0.024
Physical inactivity (%)	43.5	42.5	0.892	48.7	46.6	0.696
Currently smoke cigarettes (%)	28.4	52.2	0.003	2.8	7.1	0.110
Have family history of fracture (%)	8.7	13.1	0.216	22.4	20.5	0.637
Prior fragility fracture (%)	15.0	9.0	0.252	3.1	5.5	0.311
Body mass index (kg/m^2^)	22.76 ± 0.29	23.28 ± 0.15	0.104	23.46 ± 0.44	23.27 ± 0.12	0.671
Lean mass (kg)	45.50 ± 0.75	46.84 ± 0.37	0.099	34.38 ± 0.40	34.08 ± 0.17	0.485
Fat mass (kg)	16.34 ± 0.48	16.21 ± 0.18	0.794	18.07 ± 0.64	18.06 ± 0.20	0.991
ALP (IU/L)	264.65 ± 8.61	234.58 ± 3.18	0.001	244.25 ± 8.88	213.45 ± 3.17	0.001
25-Hydroxyvitamin D (ng/mL)	18.88 ± 0.91	20.71 ± 0.50	0.080	18.05 ± 0.68	17.09 ± 0.29	0.178
Total calorie intake (kcal/day)	2036.76 ± 119.17	2211.22 ± 48.50	0.169	1675.68 ± 60.65	1662.67 ± 27.84	0.847
Total protein intake (g/day)	70.70 ± 4.84	77.36 ± 2.39	0.213	59.17 ± 2.75	58.99 ± 1.10	0.950
Total calcium intake (mg/day)	578.86 ± 52.77	519.24 ± 23.59	0.307	511.12 ± 38.38	463.21 ± 12.74	0.239
Postmenopausal (%)	NA	NA	NA	66.6	45.3	<0.001

Numbers were showed as absolute numbers before the weighting. Data were expressed as the estimated means ± standard error or estimated percentage. P-values were for the chi-square test for categorical variables and t-test for continuous variables. A p- value of less than 0.05 was considered as statistically significant

Abbreviations: ALP, alkaline phosphatase; NA, not applicable;

We compared BMD and T-scores at various sites between cancer survivors and healthy groups after stratifying by age into those younger than 50 years, and those over 50 years of age. A statistically significant difference was found in lumbar BMD of young survivors compared with healthy groups (0.93 ± 0.04 vs. 1.02 ± 0.01 g/cm^2^, *p* = 0.032 in males; 0.95 ± 0.02 g/cm^2^ vs. 0.98 ± 0.01 g/cm^2^, *p* = 0.015 in females). There was no significant difference in the BMD of the femur neck or the total femur in cancer survivors versus healthy groups in either sex (Tables 2 and 3).

**Table 2:** Age-adjusted characteristics of bone health at various sites by aged 50 years or older in men.

	Young (<50 years)			Old (≥50 years)		
	Cancer survivors (n = 14)	Healthy comparison (n = 70)	p*-*value	Cancer survivors (n = 85)	Healthy comparison (n = 425)	p*-*value
Femur neck BMD (g/cm^2^)	0.83 ± 0.04	0.88 ± 0.01	0.179	0.72 ± 0.02	0.73 ± 0.01	0.351
Total femur BMD (g/cm^2^)	0.97 ± 0.04	1.01 ± 0.01	0.234	0.89 ± 0.01	0.91 ± 0.01	0.188
Lumbar spine BMD (g/cm^2^)	0.93 ± 0.04	1.02 ± 0.01	0.032	0.90 ± 0.02	0.93 ± 0.01	0.072
Femur neck T-score	−0.28 ± 0.29	0.29 ± 0.11	0.059	−1.03 ± 0.12	−0.91 ± 0.05	0.351
Total femur T-score	0.06 ± 0.21	0.45 ± 0.08	0.083	−0.37 ± 0.10	−0.23 ± 0.04	0.188
Lumbar spine T-score	−0.81 ± 0.28	−0.10 ± 0.11	0.020	−1.06 ± 0.12	−0.81 ± 0.07	0.072

Numbers were showed as absolute numbers before the weighting. Data were expressed as the estimated means ± standard error. The t-test was used for comparing two groups of continuous data. A p- value of less than 0.05 was considered as statistically significant.

Abbreviations: BMD, bone mineral density.

**Table 3:** Age-adjusted characteristics of bone health at various sites by aged 50 years or older in women.

	Young (<50 years)			Old (≥50 years)		
	Cancer survivors (n = 76)	Healthy comparison (n = 380)	p*-*value	Cancer survivors (n = 84)	Healthy comparison (n = 420)	p*-*value
Femur neck BMD (g/cm^2^)	0.74 ± 0.01	0.75 ± 0.01	0.452	0.66 ± 0.01	0.66 ± 0.01	0.653
Total femur BMD (g/cm^2^)	0.89 ± 0.01	0.90 ± 0.01	0.530	0.80 ± 0.01	0.81 ± 0.01	0.248
Lumbar spine BMD (g/cm^2^)	0.95 ± 0.02	0.98 ± 0.01	0.015	0.81 ± 0.02	0.83 ± 0.01	0.306
Femur neck T-score	−0.56 ± 0.12	−0.46 ± 0.05	0.461	−1.37 ± 0.10	−1.32 ± 0.05	0.653
Total femur T-score	0.34 ± 0.12	0.40 ± 0.06	0.656	−0.48 ± 0.11	−0.34 ± 0.05	0.248
Lumbar spine T-score	−0.53 ± 0.13	−0.20 ± 0.06	0.015	−1.72 ± 0.14	−1.56 ± 0.07	0.306

Numbers were showed as absolute numbers before the weighting. Data were expressed as the estimated means ± standard error. The t-test was used for comparing two groups of continuous data. A p- value of less than 0.05 was considered as statistically significant.

Abbreviations: BMD, bone mineral density.

We performed additional analyses to examine the BMD with respect to the distribution of LM and FM. Cancer survivors and healthy controls were divided into three groups according to the LM or FM, and the mean BMD was compared in each group. Table 4 shows that the BMD in the lumbar spine increased linearly with an increase of LM and FM after adjusting for potential confounding factors. There was a significant linear correlation between BMD and LM in male (p for trend < 0.001) and female survivors showed a marginal correlation between BMD and LM (p for trend = 0.060). The linear correlation between FM and BMD was significant in male cancer survivors (p for trend = 0.019) and healthy women (p for trend < 0.001) after adjustment (Table 4).

**Table 4:** Lumbar spine bone mineral density (BMD) according to group specific tertiles of lean mass or fat mass.

Lean mass*	First tertile (range, kg)	Second tertile (range, kg)	Third tertile (range, kg)	P for trend
Men, cancer survivor	(32.68–41.75)	(42.03–46.79)	(47.01–57.03)	
Fully adjusted (g/cm^2^)	0.82 ± 0.03	0.96 ± 0.02	0.97 ± 0.03	<0.001
Men, healthy	(28.24–42.70)	(42.75–47.92)	(47.94–74.35)	
Fully adjusted (g/cm^2^)	0.87 ± 0.02	0.95 ± 0.02	0.99 ± 0.02	<0.001
Women, cancer survivor	(23.96–31.91)	(31.95–35.27)	(35.28–53.12)	
Fully adjusted (g/cm^2^)	0.95 ± 0.04	0.97 ± 0.03	1.02 ± 0.04	0.060
Women, healthy	(19.04–31.73)	(31.74–35.41)	(35.45–53.64)	
Fully adjusted (g/cm^2^)	0.88 ± 0.01	0.88 ± 0.01	0.90 ± 0.01	0.249
Fat mass†	First tertile (range, kg)	Second tertile (range, kg)	Third tertile (range, kg)	P for trend
Men, cancer survivor	(3.58–10.36)	(10.75–14.73)	(14.88–25.80)	
Fully adjusted (g/cm^2^)	0.85 ± 0.03	0.91 ± 0.03	0.98 ± 0.03	0.019
Men, healthy	(2.96–10.88)	(10.90–14.63)	(14.66–39.91)	
Fully adjusted (g/cm^2^)	0.96 ± 0.02	0.94 ± 0.02	0.95 ± 0.01	0.855
Women, cancer survivor	(4.19–14.80)	(14.92–19.37)	(19.46–38.64)	
Fully adjusted (g/cm^2^)	0.98 ± 0.04	0.94 ± 0.04	0.98 ± 0.03	0.171
Women, healthy	(4.23–15.60)	(15.62–19.46)	(19.51–39.91)	
Fully adjusted (g/cm^2^)	0.85 ± 0.01	0.89 ± 0.01	0.92 ± 0.01	<0.001

Data were expressed as the estimated means ± standard error. *Adjusted for fat mass, drinking, smoking, exercise, 25(OH)D (ng/mL), height (m), daily protein intake (g/day), age groups (<50 or ≥50 years) and menopausal status (pre- or postmenopausal, in women). **†**Adjusted for lean mass, drinking, smoking, exercise, 25(OH)D (ng/mL), height (m), daily protein intake (g/day), age groups (<50 or ≥50 years) and menopausal status (pre- or postmenopausal, in women).

## Discussion

This nationwide Korean study confirms that Korean cancer survivors aged less than 50 years had lower BMD in the lumbar spine compared with healthy controls. Our results also demonstrated that LM had protective effects on lumbar BMD in adult cancer survivors as it had in healthy controls, even after consideration of covariates.

Bone density of men reached a peak when they were in their thirties and then gradually decreased, while women maintained their bone mass until their forties until it rapidly decreased in their fifties in the general population of Korea. The rate of decrease in bone mineral density varied by the measurement site; prevalence of osteoporosis of the femoral neck began to increase at 55 years of age for men and 60 years of age for women, while lumbar osteoporosis began at a younger age^13^. We found that cancer survivors have decreased lumbar bone density before their fifties, while healthy adults do not yet begin to lose lumbar bone density. High serum ALP in cancer survivors also suggests more bone turnover and loss of bone mass. The ALP level is a biochemical marker of bone turnover that is inversely related with BMD^5^. More female cancer survivors experienced menopause than healthy controls, suggesting that cancer-associated early menopause contributed to bone mineral density reduction. Since cancer survivors in our study were afflicted with various types of cancer, it is likely that the reduction in bone mineral density in cancer survivors is not limited to specific types of cancers. A six-year follow-up study of survivors of gastric cancer, a common cancer among Koreans, found that one-third of gastric cancer survivors had osteoporosis^14^. The proportion of the trabecular bone that is metabolically more active and responsive to hormones is more abundant in the lumbar spine than in the femoral neck^15,16^. Trabecular bone also has a higher turnover rate than cortical bone, and it may affect vulnerability to cytotoxic chemotherapeutic agents^17^.

Previous studies on the association between body composition and BMD suggested that LM was a positive predictor of BMD in both sexes^10,18^. Our result confirmed that a protective effect of LM on BMD was also evident in cancer survivors. However, the relationship between FM and BMD showed inconsistent results across studies with various sex and age groups. In some studies, FM was significantly associated with increased lumbar BMD, especially in postmenopausal women^16,19–21^. Another study showed that increased abdominal fat had a negative^22,23^ or null association with BMD^24^. In studies on men, FM was inversely^23^, positively^19,25^ or not correlated with BMD^26^. The adipose tissue provides a simple static load on the bone, whereas LM applies a dynamic load through muscle contraction. Thus, it is plausible that LM is more important in bone health than FM^7,27^. Excessive FM may not be beneficial for bone mass. Adipose tissues release inflammatory cytokines such as TNF-α (Tumor Necrosis Factor- α), IL-1 (Interleukin 1), and IL-6 (Interleukin 6), which lead to articular bone erosions^10,28^.

We have confirmed that young cancer survivors are at increased risk of bone loss following cancer diagnosis and its treatment. Interventions should be implemented to prevent premature fractures and minimize bone loss in young cancer survivors. Measurement of BMD can be considered to determine the bone density status of cancer survivors at younger ages than for patients among the general population. Moderate-intensity aerobic or resistance exercise may help maintain bone health after cancer treatment^29^. Our results showed that smoking and alcohol consumption by the cancer survivors were significantly lower than those of the healthy population, suggesting they chose a healthier lifestyle after a cancer diagnosis. In contrast, physical inactivity, dietary protein, and calcium intake were similar in healthy controls. Further intervention is required to prevent bone loss and increase lean mass.

Our study has several strengths. Previous studies on the association of body composition and BMD were mainly conducted on patients from the general population, and studies on cancer survivors were still limited. This is one of the few investigations that have evaluated the association of body composition and BMD in cancer survivors. It is a large population-based study of both male and female cancer survivors for an average of 6 years after a cancer diagnosis with various cancer types. Most previous studies on body composition and BMD have been limited to women who had breast cancer for several months after diagnosis^2,7,8^. However, our study included survivors with various cancers, both young and old. Our analysis of both men and women enabled us to evaluate differences in the relationship between body composition and BMD according to sex. We conducted a case-control matching analysis, wherein we assigned a healthy control to cancer survivors with case-control ratios of 1:5 by sex and age, thereby maintaining balance across important covariates between them and considering age-related changes in bone density and body composition.

Our results should be interpreted in the context of several limitations. We did not have data available to investigate the effect of cancer stage and therapeutic methods on BMD. We also did not analyze age- and sex- adjusted BMD z-scores for subjects younger than 50 years of age. Due to the cross-sectional design of the Korean National Health and Nutrition Survey (KNHANES), we did not confirm the temporal relationship. The KNHANES did not distinguish between surgical and normal menopause after removal of the uterus with or without both ovaries.

In conclusion, younger cancer survivors were at increased risk for low lumbar BMD 6 years after their cancer diagnosis compared to age- and sex-matched healthy controls without cancer history. Higher amounts of LM had beneficial effects on BMD in cancer survivors. Further research is needed to develop optimal methods of screening for osteoporosis and to discover preventive strategies that will increase lean mass and promote bone health in cancer survivors.
